# Non-canonical Translation in Plant RNA Viruses

**DOI:** 10.3389/fpls.2017.00494

**Published:** 2017-04-06

**Authors:** Manuel Miras, W. Allen Miller, Verónica Truniger, Miguel A. Aranda

**Affiliations:** ^1^Centro de Edafología y Biología Aplicada del Segura - CSICMurcia, Spain; ^2^Department of Plant Pathology and Microbiology, Iowa State UniversityAmes, IA, USA

**Keywords:** non-canonical translation, RNA structure and function, translation enhancers, translational recoding, protein synthesis, IRES, 3′-CITE

## Abstract

Viral protein synthesis is completely dependent upon the host cell's translational machinery. Canonical translation of host mRNAs depends on structural elements such as the 5′ cap structure and/or the 3′ poly(A) tail of the mRNAs. Although many viral mRNAs are devoid of one or both of these structures, they can still translate efficiently using non-canonical mechanisms. Here, we review the tools utilized by positive-sense single-stranded (+ss) RNA plant viruses to initiate non-canonical translation, focusing on *cis*-acting sequences present in viral mRNAs. We highlight how these elements may interact with host translation factors and speculate on their contribution for achieving translational control. We also describe other translation strategies used by plant viruses to optimize the usage of the coding capacity of their very compact genomes, including leaky scanning initiation, ribosomal frameshifting and stop-codon readthrough. Finally, future research perspectives on the unusual translational strategies of +ssRNA viruses are discussed, including parallelisms between viral and host mRNAs mechanisms of translation, particularly for host mRNAs which are translated under stress conditions.

## Introduction

Viruses usurp the metabolism of the host cell in their own benefit. Viral mRNA translation is a paradigmatic illustration of this, as the hallmark of viruses is that their genomes do not code for a protein synthesis apparatus. Thus, viruses have evolved many subtle ways to use and control the translational machinery of their hosts (Jiang and Laliberté, 2011; Echevarría-Zomeño et al., 2013; Walsh et al., 2013), and in fact the host range of a given virus may be determined by its ability to efficiently translate viral mRNAs using host translation factors, as we have shown recently for a plant virus (Truniger et al., 2008; Nieto et al., 2011; Miras et al., 2016). From a strategic point of view, understanding how viruses translate their own proteins may significantly contribute to the identification of therapeutic (Robert et al., 2006; Cencic et al., 2011) or breeding targets (Nicaise et al., 2003; Gao et al., 2004; Ruffel et al., 2005; Stein et al., 2005; Nieto et al., 2006; Naderpour et al., 2010). Also, understanding the peculiarities of viral mRNA translation can provide important biotechnological tools for protein overexpression (Sainsbury and Lomonossoff, 2014; Lomonossoff and D'Aoust, 2016), given the very efficient translation of some viral mRNAs in diverse conditions. From a fundamental point of view, viral mRNAs constitute powerful probes to uncover the varied and fascinating mechanisms of protein translation and their control. In this review, we describe current knowledge on the mechanisms used by positive-sense single-stranded (+ss) RNA plant viruses to initiate translation, focusing on *cis*-acting sequences present in viral mRNAs. We also describe other protein translation strategies used by plant viruses to optimize the usage of the coding capacity of their very compact genomes, including leaky scanning initiation, ribosomal frameshifting and stop-codon readthrough.

## Canonical translation of eukaryotic mRNAs

To understand the mechanisms of non-canonical translation of viral mRNAs, we first review briefly how canonical eukaryotic mRNA translation proceeds. Most eukaryotic mRNAs are appended at the 5′ end with a m^7^G(5′)ppp(5′)N cap structure, and a poly(A) tail at the 3′ end, which are critical *cis*-acting elements during canonical translation. Traditionally, translation is divided into four distinct steps: initiation, elongation, termination and ribosomal recycling. Translation initiation is the rate limiting and most highly regulated step (reviewed in Aitken and Lorsch, 2012) and begins with the formation of the 43S preinitiation complex (PIC). PIC is composed of the ternary complex (TC) eIF2-Met-tRNA-GTP bound to the 40S ribosome subunit through the P-site and the eukaryotic initiation factors (eIFs) eIF3, eIF5, eIF1A, and eIF1 (Sonenberg and Hinnebusch, 2009). EIF3, which is a large thirteen-subunit complex (Sun et al., 2011; Browning and Bailey-Serres, 2015; Smith et al., 2016), interacts with eIF2 via its subunit eIF3a and indirectly via eIF5 bridging these two factors (Valášek et al., 2002; Jivotovskaya et al., 2006). Interestingly, the eIF3d subunit can act as a cap-binding protein and is required for specialized cap-dependent translation (Lee et al., 2016).

In parallel to PIC formation, recognition of the mRNA is facilitated through binding of the cap-binding protein eIF4E to the 5' cap and the poly(A)-binding protein (PABP) to the 3′ poly(A) tail (Pestova et al., 2001). EIF4G interacts with eIF4E through its highly conserved canonical binding domain and forms, together with the helicase eIF4A, the eIF4F complex. Very recently, a second eIF4E-binding domain has been described in eIF4G, suggesting a bipartite eIF4E-eIF4G binding mode for higher eukaryotes (Grüner et al., 2016). EIF4G can also recruit other factors, including eIF3 and PABPs through direct protein-protein interactions. It is thought that the eIF4G-PABP interaction promotes the circularization of the message enhancing translation efficiency (Gray et al., 2000; Paek et al., 2015). This model is supported by biochemical data and by atomic force microscopy studies that confirm the interactions and the circularization of the mRNA (Wells et al., 1998; Kahvejian et al., 2001). However, there is increasing evidence that circularization may vary in importance for stimulation of translation among different organisms (i.e., yeast) and cells types. For example, the eIF4G-PABP interaction is not required for wild-type cell growth in yeast and mammals (Hinton et al., 2007; Park et al., 2011). Similarly, it was observed by cryo-EM that the formation of circular polyribosomes was independent of the cap structure and poly(A) tail (Madin et al., 2004; Afonina et al., 2014). These results suggest alternative mechanisms for mRNA circularization that may mimic the strategies used by +ssRNA viruses detailed in this review.

Once the mRNA is circularized, the 43S PIC in its open conformation is able to bind to the mRNA near its 5′ end. The exact mechanistic details are unknown, but eIF3 and eIF4G appear to facilitate this step (Aitken and Lorsch, 2012). The 43S PIC searches for the mRNA start codon, scanning downstream of the leader sequence resulting in the entry of the 5′ proximal start codon into the 40S subunit P-site (Kozak, 2002). Start codon selection requires cooperation between the scanning ribosome and eIF1, eIF2, and eIF5, forming the 48S preinitiation complex (Pestova and Kolupaeva, 2002). Once the start codon enters the P-site, the 60S subunit joins, with the release of eIF2, eIF1, and eIF5 and the association with eIF5B-GTP (Pestova et al., 2001). With the formation of the resulting 80S complex, the GTP molecule associated with eIF5B is hydrolyzed and released (Pestova et al., 2001).

Translation continues with the elongation phase, where the polypeptide is formed. In the elongation stage, entering amino acyl-tRNAs (aa-tRNA) bind to the A-site through the second codon of the mRNA (Lewin, 2008). After the aa-tRNA is located at the A-site, the peptidyl-tRNA is relocated from the P-site to the A-site. Once the peptide bond is formed, the translocation step occurs when the ribosome moves in a 3′ direction along the mRNA, placing a new codon at an empty A-site while the new peptidyl-tRNA is moved to the P-site and the deacylated tRNA in the E-site is ready to exit the ribosome (Julián et al., 2008; Rodnina and Wintermeyer, 2009). After the nascent polypeptide has been released, ribosomes remain bound to the mRNA and tRNA. It is only during the ribosomal recycling phase when the ribosome subunit dissociation occurs leaving them free to bind new mRNAs (Pisareva et al., 2011; Dever and Green, 2012).

## Non-canonical translation initiation of viral mRNAs

Mechanisms of non-canonical translation initiation include those that function independently of a 5′ cap or/and a poly(A) tail. These can be mediated by stimulators present in *cis* in the 5′-UTR, for example internal ribosome entry sites (IRESes) or genome-linked viral proteins (VPgs), in the 3′-UTR, for example cap-independent translation elements (3′-CITE) or tRNA-like structures (TLS), and also in intergenic regions, for example intergenic IRESes (Table 1).

**Table 1:** **Translation enhancers known in RNA plant viruses**.

**Type**	**Virus**	**Family**	**5′/3′ structures**	**Genome localization**	**Type of 3′-CITE**	**RNA secondary structure**	**eIFs/Other**	**18S RNA complementarity**	**References**
IRES	TEV	*Potyviridae*	VPg/poly(A)	5′-UTR		Two pseudoknots^a^	eIF4G	✔	Zeenko and Gallie, 2005; Ray et al., 2006
	PVY	*Potyviridae*	VPg/poly(A)			Two stem-loops^b^			Yang et al., 1997
	TuMV	*Potyviridae*	VPg/poly(A)			Stem-loop^b^	RPS6		Basso et al., 1994; Yang et al., 2009
	TriMV	*Potyviridae*	VPg/poly(A)			Two stem-loops^a^	eIF4G/eIFiso4G		Roberts et al., 2015, 2017
	BRV	*Secoviridae*	VPg/poly(A)			Stem-loop^b^			Karetnikov and Lehto, 2007
IRES	PRLV	*Luteoviridae*	5′pppN/-OH	Intergenic region		Bulge stem-loop^b^			Jaag et al., 2003
	HCRSV	*Tombusviridae*	5′pppN/-OH			Bulge stem-loop^b^			Koh et al., 2003
	PFBV	*Tombusviridae*	5′pppN/-OH			Bulge stem-loop^b^			Fernández-Miragall and Hernández, 2011
	TCV	*Tombusviridae*	5′pppN/-OH			Bulge stem-loop^b^			May et al., 2017
	crTMV	*Virgaviridae*	m^7^GpppN/TLS			None			Dorokhov et al., 2002
TLS	TYMV	*Tymoviridae*	m^7^GpppN/TLS	3′-UTR		tRNA-like^a^	eEF1a/ 40S		Matsuda et al., 2004; Colussi et al., 2015
	BMV	*Bromoviridae*	m^7^GpppN/TLS						Barends et al., 2004
CPB	AMV	*Bromoviridae*	m^7^GpppN/-OH	3′-UTR		Six stem-loops^b^	CP/eIF4G/iso4G		Neeleman et al., 2004; Krab et al., 2005
3′CITE	STNV	*Tombusviridae*	5′pppN/-OH	3′-UTR	TED	Long stem-loop^a^	eIF4F/iso4F		Meulewaeter et al., 1998; Gazo et al., 2004
	PLPV	*Tombusviridae*	5′pppN/-OH		TED	Long stem-loop^a^			Blanco-Pérez et al., 2016
	MNSV	*Tombusviridae*	5′pppN/-OH		ISS	Stem-loop^a^	eIF4F		Truniger et al., 2008; Miras et al., 2016
	MNeSV	*Tombusviridae*	5′pppN/-OH		ISS	Stem-loop^a^	eIF4F		Nicholson et al., 2010
	MNSV-N	*Tombusviridae*	5′pppN/-OH		CXTE	Two helices protruding central hub^a^	eIF4E independent		Miras et al., 2014
	BYDV	*Luteoviridae*	5′pppN/-OH		BTE	Basal helix plus 3 helices^a^	eIF4G/40S	✔	Treder et al., 2008; Sharma et al., 2015
	RSDaV	*Luteoviridae*	5′pppN/-OH		BTE	Complex BTE structure^a^	eIF4G	✔	Wang et al., 2010
	RCNMV	*Tombusviridae*	5′pppN/-OH		BTE	Basal helix plus 5 helices^a^	eIF4G	✔	Wang et al., 2010
	TNV-D	*Tombusviridae*	5′pppN/-OH		BTE	Basal helix plus 2 helices	eIF4G	✔	Shen and Miller, 2004
	TBTV	*Tombusviridae*	5′pppN/-OH		BTE	Basal helix plus 2 helices^a^	eIF4G	✔	Wang et al., 2010
	TBSV	*Tombusviridae*	5′pppN/-OH		YSS	Three helices^a^			Fabian and White, 2004
	CIRV	*Tombusviridae*	5′pppN/-OH		YSS	Three helices^a^	eIF4F/eIFiso4F		Nicholson et al., 2013
	PEMV2	*Tombusviridae*	5′pppN/-OH		PTE	Pseudoknot^a^	eIF4E		Wang et al., 2009b, 2011
	PMV	*Tombusviridae*	5′pppN/-OH		PTE	Pseudoknot^a^			Batten et al., 2006
	SCV	*Tombusviridae*	5′pppN/-OH		PTE	Pseudoknot^a^			Chattopadhyay et al., 2011
	TCV	*Tombusviridae*	5′pppN/-OH		TSS	tRNA-like^a^	60S		Stupina et al., 2008; Zuo et al., 2010
	PEMV2	*Tombusviridae*	5′pppN/-OH		TSS	tRNA-like^a^	40S/60S		Gao et al., 2013, 2014
	BRV	*Secoviridae*	VPg/poly(A)		–	Pseudoknot^b^		✔	Karetnikov et al., 2006

a *Solution probed RNA secondary structure*.

b *Predicted RNA secondary structure*.

### Enhancers located in the 5′-UTR: internal ribosome entry sites and VPgs in *Potyviridae*

The family *Potyviridae* is the largest among plant viruses with RNA genomes. The potyviral genome acts as mRNA and codes for a single polyprotein which is cleaved by viral proteases rendering 10 final functional proteins (Revers and García, 2015). Potyviral RNAs resemble those of the animal-infecting picornaviruses: they possess a small viral protein covalently bound to their 5′ ends (VPg), instead of a 5′ cap structure, and they are polyadenlylated at their 3′ ends (Adams et al., 2005). However, VPgs in different virus families differ greatly in size and function. The well-characterized VPg of *Poliovirus* (genus *Enterovirus*, family *Picornaviridae*) is only 22 amino acids (aa) long, while that of potyviruses consists of around 192 aa.

Early studies using the model potyvirus *Tobacco etch virus* (TEV, genus *Potyvirus*, family *Potyviridae*) showed that its 5′-UTR contains a sequence that was able to enhance translation 8- to 21-fold in tobacco protoplasts (Carrington and Freed, 1990). Deletion studies identified two regions in the TEV 5′-UTR including nucleotides 26-85 and 66-118 which were able to stimulate translation 10-fold with respect to a capped RNA control (Zeenko and Gallie, 2005); these regions were consequently named cap-independent regulatory elements (CIRE) 1 and 2 (Zeenko and Gallie, 2005). The TEV CIREs promoted translation of a second ORF when placed in a dicistronic reporter construct, suggesting that they were able to promote internal initiation like IRESes (Niepel and Gallie, 1999). However, the addition of a stem loop structure upstream of CIRE-1 and CIRE-2 in its natural 5′ end context reduced translation 30 and 70%, respectively, suggesting that the TEV leader might require an accessible 5′ end for ribosomal scanning (Niepel and Gallie, 1999). The TEV CIRE-1 folds into an AU-rich pseudoknot structure (PK1, nucleotides 38–75) which is essential for cap-independent translation. Interestingly, one loop of PK1 is complementary to a conserved region of the 18S rRNA and mutations in the 7 nt-complementary sequence (61-UACUUCU-67) were responsible for an approximately 80% decrease in translation compared to wild type (Zeenko and Gallie, 2005). This type of complementarity also occurs between the 18S rRNA and the sequence 4836-GAUCCU-4841 that belongs to the translation enhancer located in the 3′-UTR of *Barley yellow dwarf virus* (BYDV; genus *Luteovirus*, family *Luteoviridae*) (see Section on CITEs) and the polypyrimidine-rich tracts located in both IRES elements found in *Blackcurrant reversion virus* (BRV; genus *Nepovirus*, family *Comoviridae*) (Karetnikov and Lehto, 2007; Sharma et al., 2015), suggesting that these translation elements could recruit the 40S ribosomal subunit before loading to the 5′ end of the mRNA to start the scanning.

Early experiments using partially eIF4F depleted wheat germ extract showed that the TEV 5′-UTR conferred a competitive advantage over non-viral mRNAs which seemed to be lost when eIF4F was added back to wheat germ extract (Gallie and Browning, 2001). These results suggest that the TEV genome recruits eIF4F more efficiently than plant mRNAs when the concentration of this factor is limiting. Further analysis showed that, like for *Picornaviridae* IRESes, TEV translation is eIF4F-dependent and that eIF4G binds directly to both, the TEV 5′ leader and PK1 having a large entropic contribution (Ray et al., 2006). Moreover, the poly(A) tail functions synergistically with the TEV IRES to increase translation (Gallie et al., 1995), as also shown for animal-infecting picornaviral IRES-mediated translation (de Quinto et al., 2002; Thoma et al., 2004).

Like that of TEV, the 5′ leaders of *Potato virus Y* (PVY; genus *Potyvirus*, family *Potyviridae*), *Turnip mosaic virus* (TuMV, genus *Potyvirus*, family *Potyviridae*), and *Triticum mosaic virus* (TriMV; genus *Poacevirus*, family *Potyviridae*) (Table 1) have been shown to stimulate cap-independent translation. The 5′-UTR of PVY also contains an IRES that directs efficient translation of an ORF in a dicistronic vector (Levis and Astier-Manifacier, 1993), and IRES mapping showed that a 55 nt 3′ terminal region was fundamental for translation enhancement in tobacco protoplasts (Yang et al., 1997). The 131-nt long 5′ leader of TuMV conferred translational activity when placed upstream of a GUS reporter gene flanked at its 5′ end by a 33 nt vector-sequence (Basso et al., 1994); this RNA was able to promote translation *in vitro* to a similar level as capped mRNAs inhibiting cap-dependent translation when added in *trans* (Basso et al., 1994). The study from Yang et al. (2009) demonstrated that the TuMV RNA requires the ribosomal protein RPS6 for accumulation in *Nicotiana benthamiana*, and *RPS6* is up-regulated under TuMV infection in *Arabidopsis thaliana*. The silencing of *RPS6* abolished TuMV infection and also that of the non-related *Tomato bushy stunt virus* (TBSV; genus *Tombusvirus*, family *Tombusviridae*) (Yang et al., 2009). The TBSV viral RNA is uncapped and not polyadenylated, having no VPg. The RPS6 protein is related to other ribosomal proteins implicated in picornaviral and alphaviral infection and indispensable for *Hepatitis C virus* (HCV, genus *Hepacivirus*, family *Flaviviridae*) replication (Cherry et al., 2005; Montgomery et al., 2006; Huang et al., 2012).

It should be noted that the above reported IRESes of potyviruses may not be as strong as the IRESes of picornaviruses or HCV, for example. The 5′-UTRs of potyviruses are much shorter than the IRESes of the *Picornaviridae*, and lack strong structure or conserved sequence, and AUG triplets (Niepel and Gallie, 1999; Zeenko and Gallie, 2005). As mentioned above, an upstream stem-loop inhibited downstream translation mediated by the IRES, which lends doubt on whether it truly facilitates internal ribosome entry. Moreover, translation directed by the TEV 5′-UTR sequence from the internal position was orders of magnitude less efficient than when located at the natural 5′ end (Niepel and Gallie, 1999). Also, capped potyviral transcripts containing the 5′-UTR (including the IRES), linked to a reporter gene, translated more efficiently than uncapped transcripts (Carrington and Freed, 1990; Khan et al., 2008). These observations support the notion that conventional ribosome scanning from the 5′ end is important for efficient translation of potyviral RNAs.

One singular potyviral 5′-UTR that resembles a true animal virus-like IRES, is that of *Triticum mosaic virus* (TriMV) (genus *Tritimovirus, Potyviridae*). The exceptionally long (739 nt) 5′-UTR is much longer than that of other potyvirids and translation initiates at the 13th AUG triplet (Roberts et al., 2015). The minimal region of the TriMV leader for cap-independent translation resides in a 300-nt long sequence forming a secondary structure consisting of two long stem-loop-containing bulges. A hairpin structure at nucleotide positions 469-490 is required for cap-independent translation and internal translation initiation, and plays a role in its ability to compete with capped RNAs (Roberts et al., 2015). A unique feature of the TriMV IRES compared to those of other potyviruses is that it can mediate translation when a stem-loop structure is added upstream of the 5′ leader, thus its translation is 5′ end independent. The TriMV 5′-UTR interacts with eIF4G or eIFiso4G *in vitro*, and requires eIF4A helicase activity to mediate translation initiation (Roberts et al., 2017). These properties are true hallmarks of an IRES.

The VPg covalently attached to the 5′ end of potyviral RNAs may contribute directly to translational efficiency by interacting with translation initiation factors (Khan et al., 2008; Miyoshi et al., 2008). The addition of the TEV VPg together with eIF4F to a depleted wheat germ extract enhanced translation of an uncapped TEV RNA reporter (Khan et al., 2008). This enhancement correlated with an increase in the eIF4F-TEV RNA affinity in the presence of the VPg mediated through a direct interaction of the VPg with eIF4E. The disruption of VPg-eIF4E binding abolished stimulation of IRES-mediated translation *in vitro* (Khan et al., 2008). In contrast, TuMV VPg binds the isoform of eIF4E, eIFiso4E *in vitro* and *in vivo* (Leonard et al., 2004; Khan et al., 2008). PABP increases the binding affinity and stabilization of VPg with eIF4F or eIFiso4F in both viruses (Khan et al., 2009; Khan and Goss, 2012). Similarly to the TEV and the TuMV VPg, *Potato virus A* (PVA, family *Potyviridae*) VPg binds eIF4E and eIFiso4E and enhances viral translation in plants (Eskelin et al., 2011). Silencing of those host factors abolished PVA VPg-mediated stimulation of translation. Ribosomal protein P0 enhanced translation synergistically together with VPg and eIFiso4E and its stimulation depended on the PVA 5′-UTR (Hafrén et al., 2013). Further on, Hafrén et al. (2015) showed that viral HC-Pro and the host RNA binding protein varicose, both components of potyviral RNA granules, stimulated VPg-promoted translation of PVA.

All of the above mechanisms involve the VPg stimulating RNA translation *in trans*, leaving open the question of how the VPg specifically recognizes only the viral RNA. It is unknown whether the VPg acts *in cis* when it is covalently attached to the 5′ end, to simply replace the 5′ cap function in recruiting eIF4E and stimulating translation. The much smaller VPg of picornaviruses does not participate in translation, as polysome-associated picornaviral RNA lacks the VPg (Nomoto et al., 1977). Instead it primes picornavirus RNA synthesis (Paul et al., 1998). It is likely that the VPgs of all viruses also have this latter role, but to our knowledge, priming of RNA synthesis has not been demonstrated for the VPg of any plant virus.

The potyvirus VPg may functionally resemble the 13–15 kDa VPg of calici- and noroviruses (*Caliciviridae*) (Goodfellow, 2011). Like the potyvirus VPg, calicivirus VPg binds eIF4E (Goodfellow et al., 2005). This interaction is required for translation of *Feline calicivirus* (FCV, genus *Vesivirus*, family *Caliciviridae*) RNA, so the VPg acts as a functional analog of the cap (Goodfellow et al., 2005; Hosmillo et al., 2014; Zhu et al., 2015). In contrast, the VPg on norovirus RNA binds and requires eIF4G for translation initiation (Chung et al., 2014). This difference in factor binding may be associated with the different structures of their VPgs. While FCV and *Porcine sapovirus* (PSaV, genus *Sapovirus*, family *Caliciviridae*) VPgs adopt a compact three-helical bundle structure, *Murine norovirus* (MNV, genus *Norovirus*, family *Caliciviridae*) VPg has only two helices (Leen et al., 2013; Hwang et al., 2015). The MNV VPg-eIF4G interaction was mapped to the HEAT-1 domain in eIF4G and to the 20 C-terminal residues in VPg (Leen et al., 2016), with this latter domain differing from the eIF4E-interacting domains in FCV and PSaV VPgs. VPgs vary widely in sequence, even within a genus, so it would be difficult to extrapolate this structural information to potyvirus VPgs. Instead, to experimentally determine whether the potyvirus VPg plays the role of replacing the 5′ cap in translation, it would be valuable to determine whether translating potyvirus RNA on polysomes contains a VPg, and the effect of removing this VPg on potyvirus RNA translation.

Viruses in the family *Secoviridae* and in the genus *Sobemovirus* also have VPgs linked to their genomic RNA. The VPg of the sobemovirus *Rice yellow mottle virus* has been shown to interact with eIFiso4G and this interaction is required for viral multiplication, but a role in translation has not been published for this interaction (Hébrard et al., 2010). The role in translation of secovirids VPgs is poorly understood (Léonard et al., 2002).

### Intergenic region enhancers

IRESes have also been found in internal genomic positions within certain viral genomes (Table 1). For example, the crucifer strain of *Tobacco mosaic virus* (crTMV; genus *Tobamovirus*, family *Virgaviridae*) harbors two IRESes that stimulate the synthesis of the CP and movement protein (MP), 75 and 148-nucletotides long, respectively (Dorokhov et al., 2002, 2006). The CP IRES contains a bulged stem-loop structure that is flanked by two purine-rich repeats that are crucial for IRES activity. To find the minimal purine-rich sequence the authors reported that 16 consecutive GAAA repeats were sufficient to provide high IRES activity in plants and human cells (Dorokhov et al., 2002). However, apparently this observation has not been repeated in other labs (e.g., Fan et al., 2012). A low level of CP translation from genomic RNA of carmoviruses *Hibiscus chlorotic ringspot virus* (HCRSV) (Koh et al., 2003; Fernández-Miragall and Hernández, 2011), *Pelargonium flower break virus* (PFBV) (Fernández-Miragall and Hernández, 2011), and *Turnip crinkle virus* (TCV) (May et al., 2017) has also been reported to be IRES-mediated. Like the crTMV IRES, the TCV IRES seems to require only to be A-rich and lack of structure and its activity is inversely correlated with the size of the RNA.

Another virus that shares the crTMV polypurine tract in its IRES sequence is *Potato leafroll virus* (PLRV; genus *Polerovirus*, family *Luteoviridae*). This IRES, which is in a highly unexpected location, 22 nt downstream of the start codon and within a region of the PLRV RNA genome that is characterized by non-canonical translation mechanisms such as −1 ribosomal frameshifting, leads to translation of replication-associated protein (Rap1) (Jaag et al., 2003). The PLRV IRES element, in conjunction with the 22 nt spacer sequence, are sufficient to mediate cap-independent translation *in vitro* but not *in vivo* (Jaag et al., 2003), which sheds doubt on its biological relevance. Furthermore, this reported IRES function and the resulting translated ORF are not conserved in related poleroviruses.

Given the unstructured and sequence non-specific nature of the IRES RNA in the examples above, which is unlike the much longer, highly structured and powerful mammalian viral and dicistrovirus IRESes, we think these observations should be interpreted with caution. It may be possible that, due to lack of structure, the RNA is sensitive to nuclease cleavage providing a 5′ end, which, being unstructured, may be a very efficient leader to allow detectable translation of CP (or Rap1) ORF from undetectable amounts of degraded RNA. This alternative mechanism of expression may still be biologically relevant, or simply an artifact of the assays, but would not result from an IRES.

### Enhancers located in the 3′-UTR

#### tRNA-like structures

Viruses from the family *Bromoviridae* and the genera *Tobamovirus* and *Tymovirus* possess a 5′ cap structure but lack a 3′ poly(A) tail. In contrast, they contain tRNA-like structures (TLSs) at their 3′ termini that perform many viral processes, such as (i) serving as a telomere by interacting with CTP:ATP nucleotidyl transferase which adds CCA in a non-templated fashion to the 3′ end (Rao et al., 1989), (ii) regulation of negative strand synthesis (Dreher, 2009), (iii) translation enhancement (Gallie and Walbot, 1990; Choi et al., 2002; Matsuda and Dreher, 2004), and (iv) packaging of the viral RNA in the virion (Annamalai and Rao, 2007). Three basic types of 3′ terminal TLS have been described in the genomes of *Turnip yellow mosaic virus* (TYMV; genus *Tymovirus*, family *Tymoviridae)*, TMV and *Brome mosaic virus* (BMV; genus *Bromovirus*, family *Bromoviridae*). Because of their multiple functions, it has been difficult to tease out the mechanisms of each role, but the translational enhancement structures and mechanisms have been well characterized for TMV and TYMV (Table 1).

The TYMV TLS requires aminoacylation of the 3′-CCA terminus for maximal translational efficiency and the 5′ cap synergistically promotes this activity (Matsuda et al., 2004). Translational enhancement maps principally to the TLS, although the upstream adjacent pseudoknot is important for optimal translation, possibly serving as a sequence spacer (Matsuda and Dreher, 2004). The aminoacylated TLS binds to eukaryotic elongation factor 1A (eEF1A) and is a substrate for tRNA-modifying enzymes (Dreher and Goodwin, 1998; Matsuda et al., 2004) mimicking tRNA activity. The 5′-proximal AUG in the TYMV genome serves as start codon for a 69 kDa ORF (p69), and the second AUG is the start codon for the main polyprotein ORF (p206) with which ORF p69 overlaps. Based on only *in vitro* translation assays, Barends et al. (2004) proposed a “Trojan Horse” model of translation initiation in which the aminoacylated TLS delivers its amino acid to the start codon of the polyprotein ORF. However, the Dreher lab provided *in vitro* and *in vivo* evidence that a more likely mechanism is classical leaky scanning, except that the efficiency of initiation at the second AUG correlated with its proximity to the first AUG (Matsuda and Dreher, 2006). In addition, the translation efficiency of the polyprotein ORF depended on a 5′ cap, and not the 3′ TLS. This and additional data support an “initiation coupling” model in which the close proximity (7 nt) of the two AUG codons is necessary for maximum translation of the polyprotein ORF (Matsuda and Dreher, 2007).

How the TLS interacts with the 5′ end to stimulate translation in the scanning-dependent manner is suggested by the crystal structure of the TYMV TLS. The TLS has a tRNA-like shape, but it uses a very different set of intramolecular interactions (Colussi et al., 2015). These interactions allow the TLS to switch conformations and to interact with the ribosome, docking within it to regulate the folding and unfolding state to permit dual functionality in viral translation and replication. This leads us to hypothesize that TLS recruits the ribosome, which is delivered to the 5′-UTR by communication with the 5′ end through the cap-eIF4E-eIF4G-eIF3-40S chain of interactions.

A different function for tRNA mimicry occurs in the only IRES that occurs naturally between ORFs: the intergenic region (IGR) IRES of dicistroviruses (Wilson et al., 2000; Khong et al., 2016). In the IGR IRES, a pseudoknot mimics the structure of the anticodon loop of a tRNA basepaired to a codon in mRNA, facilitating instant elongation as the ribosome joins the viral RNA with no initiation steps (Costantino et al., 2008).

In the case of BMV RNA, its 3′-UTR has been shown to provide translation enhancement, and the disruption of its TLS reduced translation *in vitro* (Barends et al., 2004). On the other hand, the TMV TLS is structurally similar to the TYMV TLS and functions as minus-strand promoter (Chapman and Kao, 1999), but it does not mediate translation enhancement. However, the 3′-UTR of TMV contains an upstream pseudoknot domain that stimulates translation in a way that is replaceable by a poly(A) tail (Gallie et al., 1991; Leathers et al., 1993). Additionally, TMV RNA also harbors in its 5′-UTR the 68-nt omega (Ω) sequence which highly stimulates cap-dependent translation (Gallie and Kado, 1989). Ω is recognized by the heat shock protein 101 (HSP101), mediating translational activity (Wells et al., 1998) and interacts with eIF4F via eIF4G (Gallie, 2002, 2016). Similarly, the Brassicaceae-specific eIFiso4G2 isoform also contributes in Ω-mediated translation, unlike eIFiso4G which did not affect Ω-dependent translation (Gallie, 2016). These results suggest that eIFiso4G2 exhibits more functional similarity with eIF4G than eIFiso4G. Regarding translational activity, Ω is one of the most efficient mRNA leaders *in vitro* and *in vivo* and it was used for biotechnological applications such as transgene expression (Gallie et al., 1987; Fan et al., 2012).

#### 3′-UTR mediated translation of the *Alfalfa mosaic virus* genome

The non-polyadenylated *Alfalfa mosaic virus* (AMV, genus *Alfamovirus*, family *Bromoviridae*) RNA requires the viral CP for efficient translation and infection. The 3′-UTR of AMV also plays a role in translation due to its ability to bind the CP, adopting the CP-binding (CPB) conformation. This binding avoids the minus-strand promoter activity and enhances translation, possibly acting as a mimic of the poly(A) tail (Olsthoorn et al., 1999). The CPB structure folds into a series of stem-loops separated by an AUGC motif and mutations in this motif led to the loss of binding to CP, correlating with reduction of translation in protoplasts (Reusken and Bol, 1996; Neeleman et al., 2004). The crystal structure of CP-bound RNA revealed a novel RNA fold in which RNA forms two hairpins separated by the linker AUGC motif and oriented in right angles (Guogas et al., 2004). The presence of the CP promotes the base pairing between linker motifs, leading a compact structure. Moreover, pulldown assays revealed that the CP interacts with eIF4G/eIFiso4G subunits (Krab et al., 2005). This interaction may stimulate mRNA circularization in a similar fashion as found for rotaviruses (Groft and Burley, 2002). In addition to the CPB form, AMV RNAs 3′ termini also fold into a pseudoknot structure that resembles a TLS conformation. The 3′-UTR can be recognized by a tRNA-specific enzyme and by the viral replicase and this recognition is inhibited by the addition of CP (Olsthoorn et al., 1999; Chen and Olsthoorn, 2010). Thus, it suggests that TLS conformation acts as a minus-strand promoter and the CP interaction and pseudoknot stability may regulate a conformational switch between translation and replication (Chen and Olsthoorn, 2010).

#### Cap-independent translation elements

Members of the *Tombusviridae* and *Luteoviridae* plant virus families lack both 5′ cap and 3′ poly(A) elements, but contain in their 3′ ends structured RNA elements capable of enhancing translation in the absence of cap (cap-independent translation elements, CITEs). Most 3′-CITEs have in common their ability to bind translation initiation factors of the eIF4E or eIF4G families, as well as the presence of small sequence stretches within or near the 3′-CITE capable of base-pairing to sequences in the 5′-UTR of the mRNA to establish long-distance RNA:RNA interactions (Table 1). By definition, 3′-CITEs functionally substitute for the 5′ cap with high efficiency. They recruit translation initiation factors leading to ribosome entry at or near the 5′ terminus followed by ribosome scanning to the initiation codon (Fabian and White, 2004; Rakotondrafara and Miller, 2008; Nicholson and White, 2011); therefore, in contrast to IRESes, 3′-CITEs do not promote internal ribosome entry. To date, seven different classes of 3′-CITEs have been described (Simon and Miller, 2013; Miras et al., 2014) which share little secondary structure and sequence similarity. Due to space limitations and a previous comprehensive review on 3′-CITEs (Simon and Miller, 2013), we will describe only briefly each 3′-CITE and recent updates.

The first 3′-CITE was discovered in *Satellite tobacco necrosis virus* (STNV) and is located in a 120-nt sequence termed translation enhancer domain (TED) (Danthinne et al., 1993; Timmer et al., 1993; Meulewaeter et al., 1998). The TED is predicted to form a long stem-loop with several internal bulges (Van Lipzig et al., 2002). This element was shown to be functional in enhancing translation *in vitro* and *in vivo*. TED binds eIF4F or eIFiso4F (Gazo et al., 2004), and is proposed to interact with the 5′-UTR via a predicted RNA:RNA long-distance interaction with the apical loop of the 5′ end. However, mutations that disrupted this potential long-distance base-pairing reduced translation only slightly, and covarying mutations designed to restore base pairing did not restore translation to wild type levels (Meulewaeter et al., 1998). The STNV 3′-CITE confers cap-independent translation *in vitro* when it is moved to the 5′-UTR of an uncapped reporter (Meulewaeter et al., 1998). Another member of the *Tombusviridae* family, *Pelargonium line pattern virus* (PLPV, genus *Carmovirus*) was recently shown to harbor a 3′-CITE in the TED class (Blanco-Pérez et al., 2016). In this case, PLPV TED was shown to require a long-range RNA:RNA kissing stem-loop interaction with a hairpin in the coding sequence of the PLPV p27 ORF for efficient translational activity (Blanco-Pérez et al., 2016).

The shortest CITEs are the I-shaped structures (ISS) present in the 3′-UTRs of *Maize necrotic spot virus* (MNeSV, *Tombusvirus*, family *Tombusviridae*) and *Melon necrotic spot virus* (MNSV, genus *Carmovirus*, family *Tombusviridae*) (Truniger et al., 2008; Nicholson et al., 2010; Miras et al., 2016), and are apparently similar in secondary structure to the TED. MNeSV ISS has been shown to preferentially interact with the eIF4E subunit of eIF4F. As for TED and most other CITEs, base pairing between the 3′-CITE and the 5′-UTR is predicted to deliver the translation factor to the 5′ end, facilitating recruitment of the 43S preinitiation complex (Nicholson et al., 2010). In support of this model, it has been shown that the interacting 5′-UTR:I-shaped 3′-CITE of MNeSV together with eIF4F form a complex *in vitro*. In addition, ribosome toe printing demonstrated that while bound to eIF4F, the I-shaped CITE can simultaneously base pair with the 5′-UTR and recruit ribosomes to the 5′ end of the viral fragment (Nicholson et al., 2010).

In the case of MNSV ISS, genetic evidence for interaction of the ISS with eIF4E has been shown in melon. A single amino acid change in melon eIF4E strongly reduces translation efficiency controlled by MNSV ISS and makes melon resistant to MNSV infection (Nieto et al., 2006; Truniger et al., 2008). The minimal 3′-CITE sequence, named Ma5TE (MNSValpha5-like translation enhancer), was mapped to a 45 nt region. *In vitro* binding assays revealed that Ma5TE forms a complex with eIF4F and this interaction was mapped to a conserved guanosine residue located in a Ma5TE internal loop (Miras et al., 2016). Additionally, mutational analyses in eIF4E residues involved in its interaction with eIF4G showed that eIF4F complex formation is necessary for efficient cap-independent translation driven by Ma5TE (Miras et al., 2016). Identification of a new resistant-breaking isolate of MNSV revealed a new class of 3′-CITE, the CXTE, which was acquired from *Cucurbit aphid-borne yellows virus* (CABYV, genus *Polerovirus*, family *Luteoviridae*) Xinjiang by interfamilial recombination, conferring to the recipient MNSV isolate the advantage to translate efficiently and infect resistant melon varieties (Miras et al., 2014). Thus, the 3′-UTR of this MNSV isolate harbors two 3′-CITEs, Ma5TE, and CXTE, with CXTE secondary RNA structure folding into two helices protruding from a central hub. Both 3′-CITEs are active in susceptible melon, while only the CXTE functions in resistant melon and in the absence of eIF4E (Miras et al., 2014).

**Figure 1:**
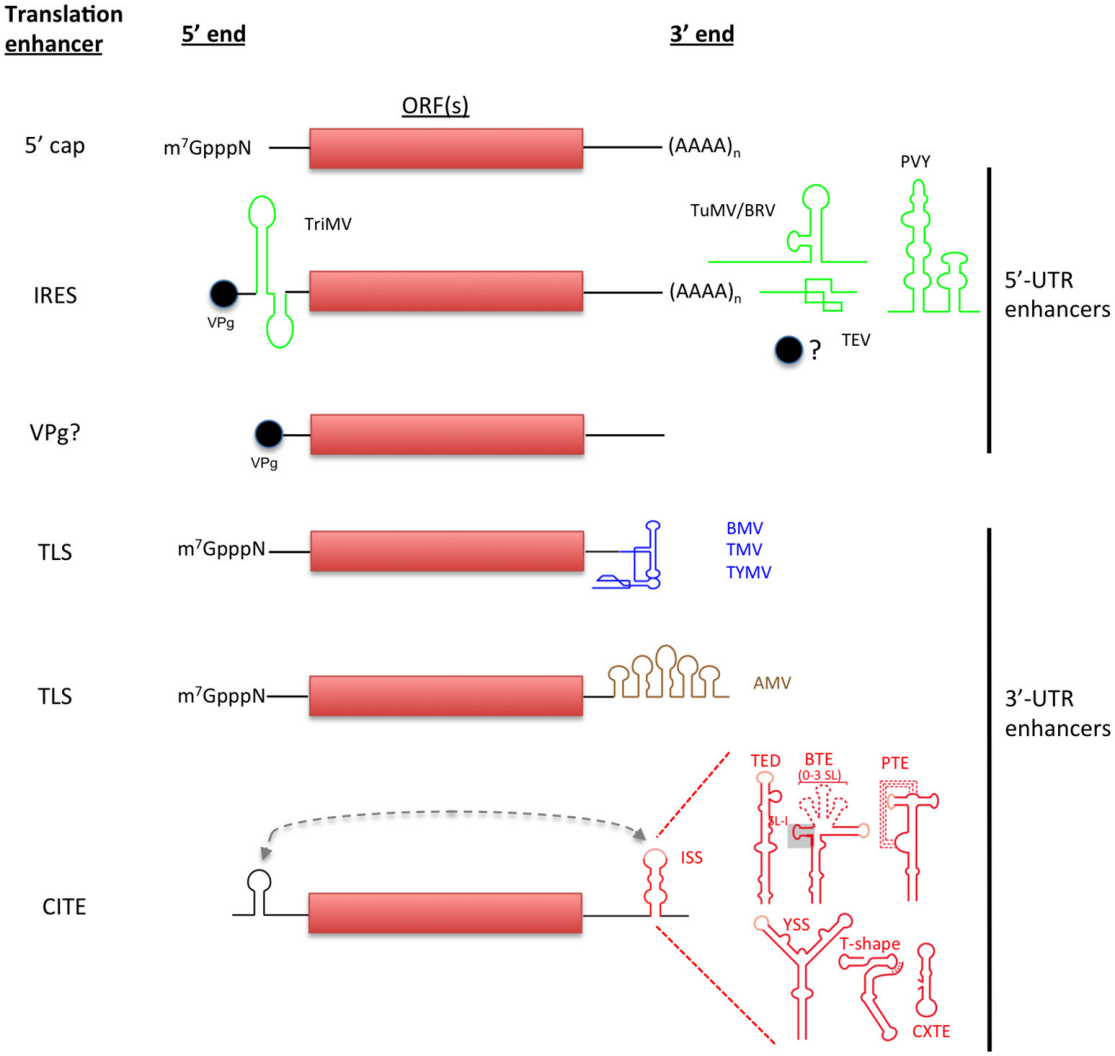
**Non-canonical initiation translation mechanisms used by plant RNA viruses**. Canonical translation of eukaryotic mRNAs is shown in the top. Non-canonical translation elements are grouped depending on their location in viral genome and are color-coded to match with the virus acronyms. Lighter-shaded loops in the secondary structure of 3′-CITEs indicated sequences known or predicted to base-pair to the 5′ end of the viral genome (shown as dashed line).

The *Barley yellow dwarf virus*-like translation element (BTE) is one of the best-characterized 3′-CITEs and is found in all members of the *Luteovirus, Dianthovirus, Alphanecrovirus, Betanecrovirus*, and *Umbravirus* genera (Wang et al., 2010; Simon and Miller, 2013). All BTEs share a long basal helix from which two to five additional helices radiate (Figure 1). BTEs contain a highly conserved 17-nucleotide sequence GGAUCCUGGGAAACAGG that includes SL-I (formed by pairing of underline bases). The BTE binds preferentially to the eIF4G subunit of the eIF4F heterodimer (Treder et al., 2008). The eIF4G-binding site in the BTE was revealed by SHAPE footprinting, which showed that eIF4G protects SL-I and nearby bases around base of the hub from which all helices protrude (Kraft et al., 2013). Addition of eIF4E enhanced the level of protection and stimulated translation by about 25%. Deletion analysis of eIF4G revealed that only the core domain (including eIF4A and eIF3 binding sites, but lacking the eIF4E and PABP binding sites) and an adjacent upstream RNA binding domain are necessary for binding to the BTE and to stimulate translation (Kraft et al., 2013; Zhao et al., 2017).

A long-distance kissing stem-loop interaction between a loop in the BTE and the 5′-UTR is required for BTE-mediated translation (Guo et al., 2001). This long-distance RNA:RNA interaction can be replaced by complementary non-viral sequences outside the BTE (Rakotondrafara et al., 2006). This interaction is conserved among all BTEs except the BTE of *Red clover necrotic mosaic virus* (RCNMV, genus *Dianthovirus*, family *Tombusviridae*), in which mutations in potential complementary loops had no effect on translation and possess the longest BTE and 3′-CITE (Sarawaneeyaruk et al., 2009).

**Figure 2:**
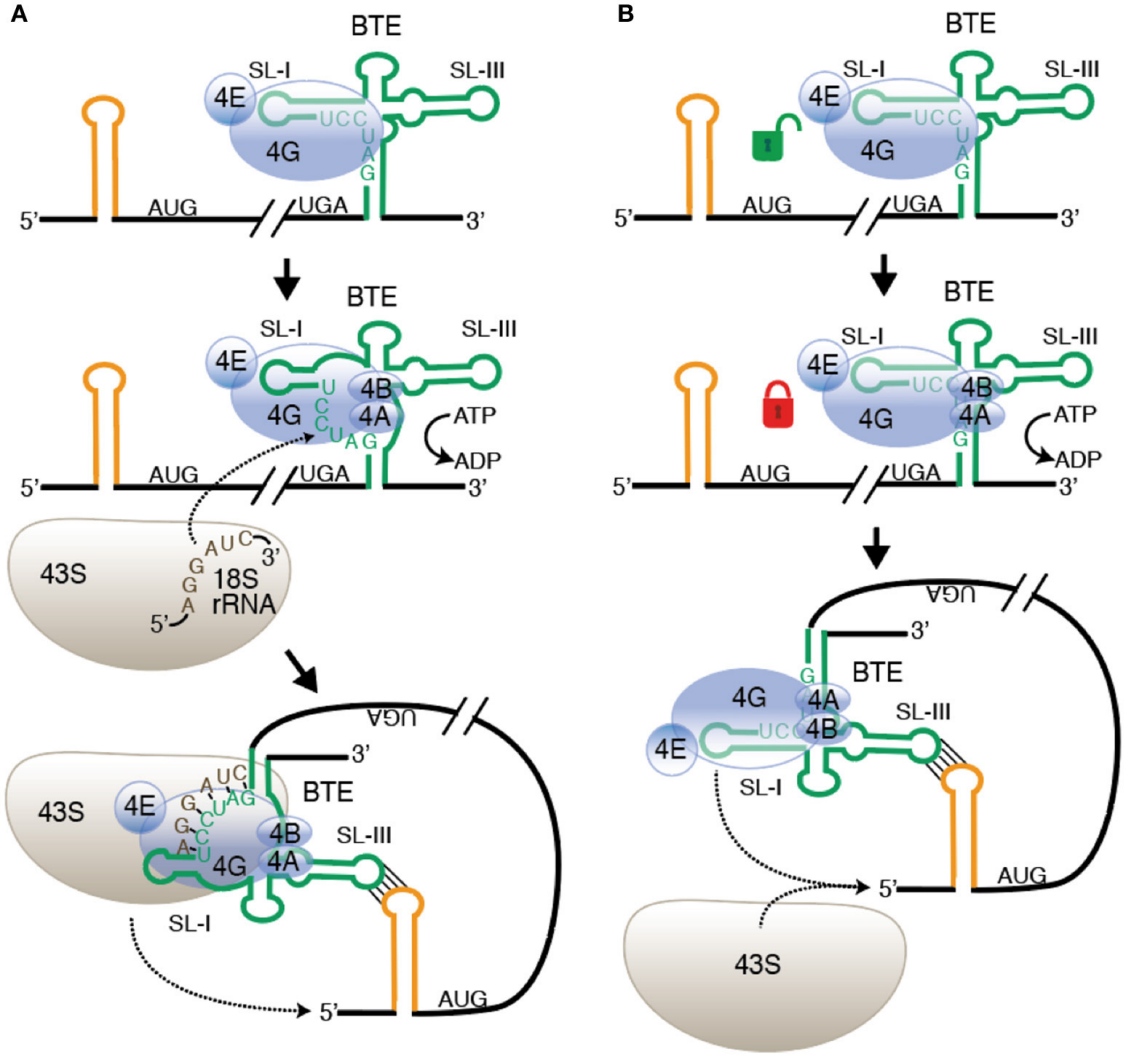
**Alternative models of ribosome recruitment and delivery to the 5′-UTR via the BTE. (A)** Base pairing to rRNA model. *Top:* eIF4F binds to SL-I of the BTE (green) through the eIF4G subunit. eIF4E enhances but is not required for BTE binding. *Middle:* Helicase (eIF4A + eIF4B) binds and uses ATP hydrolysis to unwind GAUCCU, making it available to base pair to 18S rRNA at a conserved sequence in the region where the Shine-Dalgarno binding site is located in prokaryotic 16S rRNA. *Bottom:* The 43S preinitiation complex base pairs to the BTE and is delivered to the 5′ end by long-distance base pairing (yellow stem-loop). **(B)** Conventional ribosome recruitment model. *Top:* eIF4F binds BTE as in **(A)**. *Middle:* Binding of eIF4A + eIF4B and ATP hydrolysis increases binding affinity of eIF4F, “locking” it on to the BTE, perhaps by altering the structure of BTE RNA. *Bottom*: eIF4 complex is delivered to 5′ end by long-distance base pairing where it recruits the 43S preinitiation complex to the RNA. In both models, 43S scanning from the 5′ end to the start codon is the same as in normal cap-dependent translation. *Not shown:* other factors, such as eIF3 and factors in the preinitiation complex.

After eIF4F binds the BTE, it appears that the eIF4A helicase, eIF4B plus ATP bind in order to recruit the 40S subunit directly to the BTE. The long-distance base pairing would then deliver the 40S complex to the 5′ end for scanning to the first AUG (Sharma et al., 2015; Figure 2A). This differs from a previous model in which it was proposed that the long-distance base pairing places the factors near the 5′ end, at which point the 40S complex is recruited (Rakotondrafara et al., 2006). However, the dependence on helicase activity may support an older model in which a six base tract in the 17 nt conserved sequence (GAUCCU) base pairs directly to 18S rRNA at the position where the Shine-Dalgarno sequence is located in prokaryotic ribosomal RNA (Wang et al., 1997). Because much of this tract is base paired internally in both the BTE and in 18S rRNA, the helicase activity may be required to disrupt this base pairing, freeing the complementary tracts in the BTE and 18S rRNA to base pair to each other. This base pairing would recruit the 40S subunit directly to the BTE (Figure 2A). However, recently the presence of eIF4A, eIF4B and ATP was also found to enhance the binding affinity of the BTE to eIF4G in the absence of the ribosome (Zhao et al., 2017). This enhanced binding affinity may be the consequence of helicase activity of eIF4A/eIF4G/ATP altering BTE structure. This greater affinity of eIF4G to the BTE may facilitate efficient recruitment of the 40S subunit by conventional factor interactions without need for base pairing to ribosomal RNA (Figure 2B). Future experiments are necessary to determine which model is correct. On the other hand, RCNMV possesses an A-rich sequence (ARS) with strong affinity to PABP in addition to its BTE in its 3′-UTR. Both sequences, ARS and 3′-CITE, have been shown to coordinately recruit eIF4F/ eIFiso4F and the 40S ribosomal subunit to the viral RNA (Iwakawa et al., 2012).

*Tomato bushy stunt virus* (TBSV, genus *Tombusvirus*, family *Tombusviridae*) and other viruses belonging to the genus *Tombusvirus*, contain 3′-CITEs resembling Y-shaped structure (YSS), formed by three helical regions. The efficiency of translation controlled by the YSS of TBSV depends on a long-distance interaction with the 5′-UTR of the genome. Mutational analysis of TBSV YSS showed that alterations in junction residues between helices and in a large asymmetric bulge in the major supporting stem disrupted translation (Fabian and White, 2004, 2006). Moreover, the YSS of *Carnation Italian ringspot virus* (CIRV, genus *Dianthovirus*, family *Tombusviridae*) requires addition of the eIF4F or eIFiso4F complex to a factor-depleted wheat germ extract to promote efficient translation (Nicholson et al., 2013). Translation assays showed the ability of the CIRV YSS to function efficiently *in vitro* and *in vivo*, whereas TBSV YSS was detectable only in *in vivo*, suggesting that this difference is due to a misfolding in the TBSV RNA and the lack of eIFs required in translation (Fabian and White, 2004).

The *Panicum mosaic virus*-like Translation Enhancer (PTE) was first identified in *Panicum mosaic virus* (PMV, genus *Panicovirus*, family *Tombusviridae*) (Batten et al., 2006) and later in *Pea enation mosaic virus 2* (PEMV2, genus *Umbravirus*, family *Tombusviridae*) (Wang et al., 2009b). The PEMV2 PTE consists of a three-way branched helix with a large G-rich bulge in the main stem (Wang et al., 2009b). The formation of a magnesium-dependent pseudoknot between the G-rich bulge and a C-rich sequence at the three-helix junction of the PTE is critical for translation and eIF4E recruitment by the PTE (Wang et al., 2011). Unlike most other CITEs, the PEMV2 PTE may not participate in a long-distance RNA:RNA interaction with the 5′-UTR. Instead, upstream of the PTE, there is an element, the kl-TSS, that participates in a long range RNA:RNA interaction with a 5′ proximal hairpin located in the p33 ORF (Gao et al., 2012).

Most other PTEs contain a loop predicted to base pair to the 5′-UTR. Indeed, *Saguaro cactus virus* (SCV, genus *Carmovirus*, family *Tombusviridae*), harbors a PTE which participates in a long-distance RNA:RNA interaction with a hairpin located in the p26 ORF (Chattopadhyay et al., 2011). Interestingly, the sequence involved in the interaction has the same conserved motif found in carmovirus TED-like elements and I-shaped structures (Simon and Miller, 2013).

The 3′-UTR of another member of the *Tombusviridae* family, *Turnip crinkle virus* (TCV, genus *Carmovirus*, family *Tombusviridae*), contains an internal T-shaped structure (TSS) that consists of three hairpins, two pseudoknots and multiple unpaired single stranded linker regions (Zuo et al., 2010). Interestingly, the TSS resembles a three-dimensional tRNA-like structure (Zuo et al., 2010). The TCV TSS recruits and binds the 60S subunit of the 80S ribosome (Stupina et al., 2008). For this element, no base pairing between 3′-CITE and 5′-UTR has been identified. It was proposed that the ribosomal subunits form a protein bridge with the UTRs, where the 40S subunit binds the 5′-UTR and the 60S subunit binds the TSS (Stupina et al., 2008). Two additional TSSs were found in the PEMV2 3′-UTR, one upstream of the PTE and another near to the 3′ terminus (Gao et al., 2013). Interestingly, both TSSs can also bind the 60S ribosomal subunit and although they are essential for virus accumulation *in vivo*, mutations that disrupted the downstream TSS had no effect in translation (Gao et al., 2013, 2014). However, when this TSS element was positioned proximal to the reporter ORF enhanced translational activity. This report points out the importance of the reporter constructs in the identification of 3′-CITE that participate in translation. A recent report showed that TCV RdRp binds to A-rich sequence upstream of the TSS and using optical tweezers and steered molecular dynamic simulations showed that elements of TSS unfold when it is interacting with RdRp which may promote the conformational switch between translation and replication (Le et al., 2017).

More classes of 3′-CITE await discovery, as the 3′ UTRs of several members of the *Tombusviridae* contain no structure that obviously resembles a known 3′-CITE (Simon and Miller, 2013). Thus, viruses have evolved a plethora of structures to achieve the same goal: recruitment of eIF4F and ultimately the ribosome to their RNAs.

### 5′- and 3′-UTR dependent translation of nepovirus genomic RNAs

As mentioned above, nepovirus (family *Secoviridae*, order *Picornavirales*) genomes contain a VPg linked to their 5′ end, thus are uncapped but polyadenylated requiring also cap-independent translation mechanisms. The two genomic RNAs (gRNA) of *Blackcurrant reversion virus* (BRV; genus *Nepovirus*, subfamily *Comoviridae*), have translation enhancing sequences in their 5′- and 3′-UTRs. The 5′ leader sequences of the two gRNAs of BRV contain IRES elements that facilitate translation when placed either at the 5′-end of a non-capped reporter RNA or internally between two reporter genes (Karetnikov and Lehto, 2007, 2008). The BRV IRESes contain little secondary structure, harboring only one predicted single stem-loop structure at the 5′ end. Also, the 5′-UTRs of both gRNAs have at least six AU-rich tracts of 8–10 nt predicted to base pair to 18S rRNA. Deletion of these sequences reduced cap-independent translation activity, suggesting a disruption of the required complementarity or other 5′-UTR functional features (Karetnikov and Lehto, 2007, 2008).

In addition to these IRESes, CITE activity was mapped to the 3′-UTRs of BRV RNA1 and RNA2 (Karetnikov et al., 2006; Karetnikov and Lehto, 2008). This activity depended on the presence of a predicted stem-loop structure located immediately downstream of the last ORF. Moreover, translation efficiency was shown to be dependent on a long-distance RNA interaction with a stem-loop structure present in the 5′-UTR (Karetnikov and Lehto, 2008). Secondary structures of the 3′-CITE and 5′-UTR have not been determined, although they are predicted to fold as a pseudoknot and a stem-loop, respectively. Presence of a poly(A) tail (which is naturally present in the BRV RNA, unlike in other 3′-CITE-containing viruses) in reporter mRNAs stimulated translation several fold, thus playing a major role in CITE-mediated translation. Many of the key elements identified in BRV RNAs, including 5′–3′-UTR RNA interactions and sequence complementarity with the 18S rRNA in the 5′-UTR, are predicted to be conserved in the RNAs of other nepoviruses (Karetnikov and Lehto, 2008), although their precise biological functions remain unknown.

## Optimization of coding capacity

RNA viruses often contain overlapping genes, which allows a very efficient use of the sequence to maximize the coding capacity. Expression of these overlapping genes is achieved by (i) initiation of translation at multiple start codons in different reading frames, by leaky scanning of ribosomes, (ii) frameshifting by a portion of the ribosomes during the elongation phase of translation, or (iii) generating subgenomic mRNAs that allow translation of each ORF from a separate mRNA. The latter will not be discussed, as it is not a translational control mechanism.

### Leaky scanning

**Figure 3:**
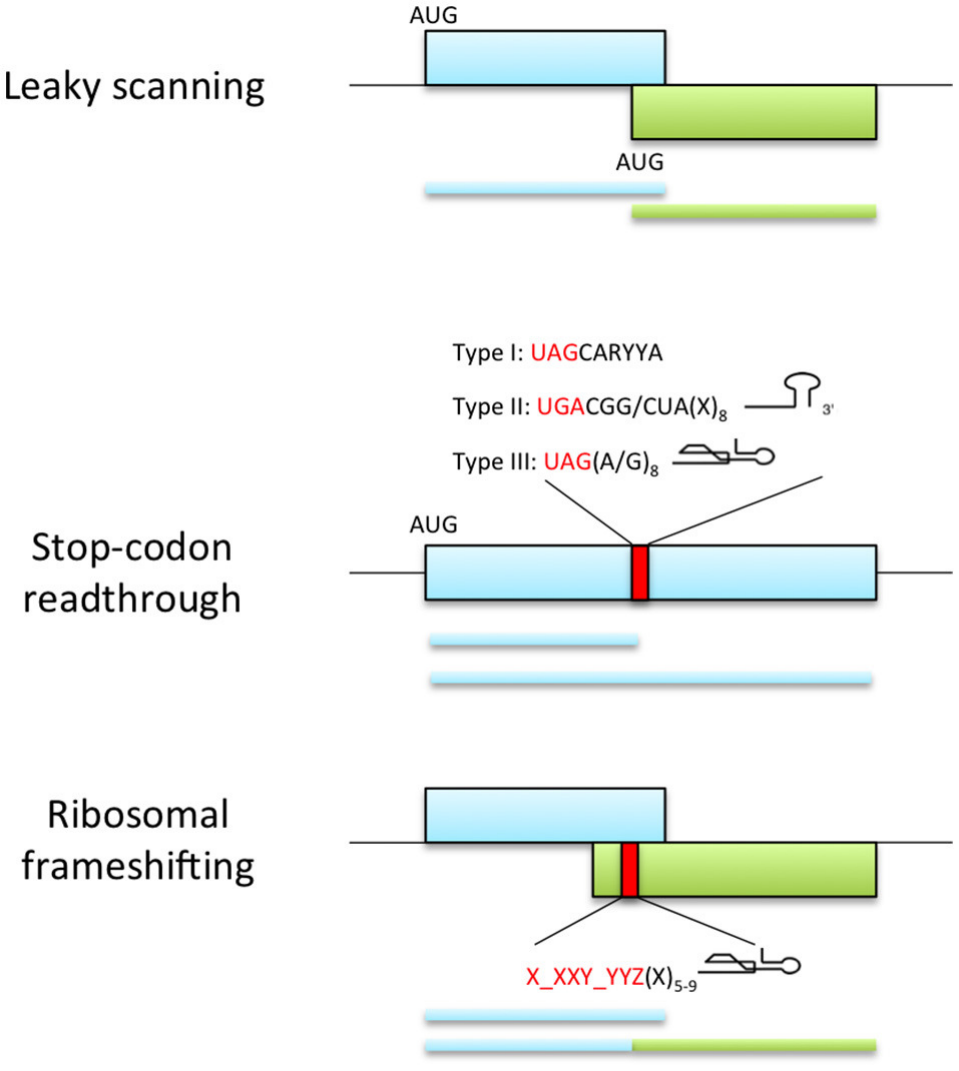
**Viral recoding strategies**. Top panel represents leaky scanning mechanism where ribosomes fail to start translation at the first AUG codon and continue scanning until they reach an alternative start codon in the optimal initiation context. This process allows the expression of two proteins with distinct amino acid sequence when the initiation sites are in different reading frames (as shown) or C-terminally coincident isoforms of a single protein if initiation sites are in-frame (not shown). Middle panel shows the expression of proteins with alternative C-terminal because a portion of ribosomes fail to terminate at a stop codon and continue translation. The efficiency of readthrough can be stimulated by the presence of elements downstream of the stop codon: UAG stop codon followed by the consensus motif CARYYA, where R is a purine and Y is a pyrimidine (Type I); UGA stop codon followed by CGG or CUA triplet and a stem-loop structure separated from the stop codon by 8 nt (Type II); UAG stop codon and adjacent G or purine octanucleotide and a compact pseudoknot structure (Type III). Bottom panel represents ribosomal frameshifting strategy, where ribosomes are directed into a different reading frame guided by the slippery signal X_XXY_YYZ (X and Y can be any base and Z is any base except G) and a secondary structure element located 5-9 nt downstream the slippery sequence.

Leaky scanning occurs when a proportion of ribosomes fail to initiate translation at the first AUG codon and continue downstream until they reach an AUG codon in the optimal caA(A/C)aAUGGCg initiation context (Figure 3; Joshi et al., 1997; Kozak, 2002). If the two AUG codons are in the same reading frame, the protein derived by initiation at the second AUG is an N-terminally truncated version of that made by initiation at the first AUG. If the two AUGs are in different frames, then the two proteins have entirely different amino acid sequences. Examples of the latter are long overlaps of replication genes and the triple gene block (TGB) that encodes the movement proteins of several viruses: For instance, the TGB3 of *Potato virus X* (PVX, family *Flexiviridae*, genus *Potexvirus*) and *Barley stripe mosaic virus* (BSMV, family *Virgaviridae*, genus *Hordeivirus*) and the TGB2 of *Peanut clump virus* (PCV, family *Virgaviridae*, genus *Pecluvirus*) are expressed by leaky scanning (Herzog et al., 1995; Zhou and Jackson, 1996; Verchot et al., 1998). In addition, leaky scanning may also be facilitated by the use of non-AUG initiation codons, which require a strong initiation context (Kozak, 1989). In this respect, *Shallot virus X* (ShVX; family *Flexiviridae*, genus *Allexivirus*) contains a non-canonical ORF for its TGB3 protein (Kanyuka et al., 1992; Lezzhov et al., 2015). ShVX TGB3 translation initiates in a CUG triplet, which has been shown previously to be the most efficient non-AUG initiator (Firth and Brierley, 2012). This triplet and flanking sequences give an optimal context for translation initiation and are conserved in all allexviruses (Lezzhov et al., 2015). Similarly, translation of the second movement protein from *Pelargonium line pattern virus* (PLPV, family *Tombusviridae*, genus *Pelarspovirus*) and *Maize chlorotic mottle virus* (MCMV, family *Tombusviridae*, genus *Machlomovirus*) were suggested to be initiated in a GUG or CUG start codons, accomplished by leaky scanning (Scheets, 2000; Hernández, 2009).

In the main subgenomic RNA of poleroviruses and luteoviruses all three reading frames are used. The tiny, 45 codon first ORF, which encodes a long-distance movement protein, always starts with a non-AUG codon, such as GUG, CUG or AUU. Thus, most scanning 40S ribosomes skip this codon (Smirnova et al., 2015). The second ORF, which encodes the coat protein, starts with AUG in a poor context, while the third ORF, a movement protein gene, starts with AUG in a strong context. The secondary structure encompassing these two AUGs also affects initiation preference (Dinesh-Kumar and Miller, 1993). Other examples of leaky scanning in replicase ORFs have been described. In tymoviruses, the first AUG initiates an ORF encoding a 69 kDa protein that overlaps with the main replicase-encoding ORF initiated by the second AUG. While Kozak context plays a role, unlike “conventional” leaky scanning, the second AUG must be in close proximity (e.g., 7 nt) of the first to efficiently initiate translation (Matsuda and Dreher, 2006). Recently, a small ORF in the sobemoviruses, ORFx, was discovered that overlaps ORF2a and is essential for *Turnip rosette virus* (TRoV, genus *Sobemovirus*) to establish systemic infection (Ling et al., 2013).

### Translational recoding: frameshift and readthrough

Recoding consists of the redefinition of individual codons in response to signals in an mRNA. Such signals could be RNA secondary structures, complementary interactions with ribosomal RNA or alteration of the ribosomal state (Atkins and Baranov, 2010). In ribosomal frameshifting a proportion of translating ribosomes are guided into a different reading frame by induced slippage of the ribosome by the mRNA structure (exhaustively reviewed by Miller and Giedroc, 2010; Atkins et al., 2016), while in readthrough mechanisms, a portion of ribosomes fail to terminate at a stop codon and continue translation (Figure 3). This generates proteins with alternative C-termini. Viruses use often these processes to express the RNA-dependent RNA polymerase domain of the replicase.

#### Ribosomal frameshifting

Many plant viruses utilize programmed ribosomal frameshifting (PRS) to translate overlapping ORFs. This recoding event can occur in the + or − direction relative to the normal 0 frame of mRNA translation by shifting the ribosome in one or two nucleotides forward or backward. Productive frameshifting normally competes poorly with standard decoding, so the efficiency of frameshifting in viruses varies from 1% in BYDV to 82% in cardioviruses (Barry and Miller, 2002; Finch et al., 2015). Thus far, most frameshifting by plant viruses is in the -1 direction. These include members of the *Sobemovirus, Umbravirus*, and *Dianthovirus* genera and the *Luteoviridae* family (Brault and Miller, 1992; Demler et al., 1993; Kujawa et al., 1993; Mäkinen et al., 1995; Kim et al., 1999; Lucchesi et al., 2000; Barry and Miller, 2002; Tamm et al., 2009). Members of the non-related family *Closteroviridae* (genus *Closterovirus, Crinivirus* and *Ampelovirus*) are predicted to use a +1 frameshift to synthesize their viral replicases (Agranovsky et al., 1994; Karasev et al., 1995; Melzer et al., 2008).

The -1 PRS usually requires two signals in the mRNA, a slippery sequence of the type X_XXY_YYZ, where X normally represents any nucleotide, Y represents A or U and Z represents A, C or U (gaps delimit codons in the original 0 frame); and a downstream secondary structure element separated from the slippery sequence by a spacer region of 5-9 nt (Dinman, 2012). In plant viruses these structural elements, acting as stimulators of frameshifting, fall into three structural classes: an apical loop with a bulge, a compact hairpin-type pseudoknot or a stem-loop (Figure 3) (reviewed by Miller and Giedroc, 2010).

The -1 PRS stimulatory elements of BYDV, PEMV-RNA2 and RCNMV fold into a stem-loop with an internal bulge in a similar manner (Kim et al., 1999; Paul et al., 2001; Barry and Miller, 2002; Gao and Simon, 2015). For BYDV, this element participates in a long-distance interaction with the apical loop of a stem-loop located in the 3′-UTR (about 4 kb downstream of the frameshift site). This interaction is required for the low expression levels of RdRp and thus replication (Barry and Miller, 2002). Similar long-range base pairing interactions were shown in RNAs of RCNMV and PEMV2 (Tajima et al., 2011; Gao and Simon, 2015). For PEMV2 RNA, this interaction modifies the lower stem of the structure, possibly due to a rise of its stability or the approximation of other sequence near the 3′ end. Curiously, the distant −1 PRS element of PEMV2 RNA appeared to inhibit, rather than stimulate frameshifting, because in its absence, the frameshift rate increased 72% with respect to the wild type viral genome (Gao and Simon, 2015).

On the other hand, the frameshift stimulatory elements from poleroviruses *Beet western yellows virus, Potato leaf roll virus* and *Sugarcane yellow leaf virus* (BWYV, PLRV, and ScYLV, family *Luteoviridae*, genus *Polerovirus*) and PEMV1 (family *Luteoviridae*, genus *Enamovirus*) form h-type pseudoknots (Egli et al., 2002; Cornish et al., 2005; Pallan et al., 2005; Giedroc and Cornish, 2009). The frameshift regulatory element of BWYV was the first to be determined at atomic resolution showing a compact pseudoknot with a triple-stranded region (Egli et al., 2002). It was suggested that pseudoknots provide a kinetic barrier to the ribosome and that the unfolding of this element correlates with frameshifting stimulation (Giedroc and Cornish, 2009).

#### Stop-codon readthrough

Stop-codon readthrough is a common strategy found in plant viruses to encode protein variants with an extended C-terminus from the same RNA. During readthrough, some ribosomes do not stop at the stop codon but continue until the next termination codon. Members of the *Tombusviridae, Luteoviridae* and *Virgaviridae* families employ readthrough of UGA and UAG stop codons in their replicase and coat protein genes. Flanking nucleotides as well as long-range RNA-RNA interactions influence stop-codon readthrough (Figure 3; Firth and Brierley, 2012; Nicholson and White, 2014). Depending on the sequence motifs and the stop codon, three types of readthrough can be described: The type I motif employs a UAG codon in the replicase gene and is followed by the consensus motif CARYYA (where R is a purine and Y is a pyrimidine) (Skuzeski et al., 1991); this type is used by tobamoviruses, benyviruses and pomoviruses (Pelham, 1978; Firth and Brierley, 2012). The type II motif is used by tobraviruses, pecluviruses, furoviruses and pomoviruses to generate their viral RdRp and by furoviruses to express the coat protein (Skuzeski et al., 1991; Zerfass and Beier, 1992). It involves a UGA stop codon followed by a CGG or CUA triplet and a stem-loop structure about 8 nts downstream of the stop codon (Firth et al., 2011). The type III class comprises an UAG stop codon, a downstream G or purine-rich octanucleotide and a 3′ RNA structure (Firth and Brierley, 2012) and appears in carmovirus and tombusvirus genomes. For example, the tombusvirus CIRV uses stop-codon readthrough to generate its viral RdRp and requires a long-distance interaction between an RNA structure located downstream of the readthrough site and also a sequence in the 3′-UTR (Cimino et al., 2011). *Tobacco necrosis virus-D* (TNV-D, genus *Betanecrovirus*, family *Tombusviridae*) employs a complex series of downstream interactions. A stable bulged readthrough stem-loop (RTSL) immediately downstream of the leaky stop codon contains a G-rich bulge which must base pair to a distant readthrough element (DRTE) located 3 kb downstream in the structure required for replication initiation (Newburn et al., 2014). A pseudoknot immediately 3′ to the RTSL, and a stem-loop adjacent 5′ to the DRTE in the 3′-UTR are also necessary for optimal readthrough (Newburn and White, 2017). The long-distance interactions within the viral genome required for frameshifting and readthrough may play a regulatory role as switch between translation and replication (Cimino et al., 2011), by allowing replicase entering at the 3′ end of the genome to stop its own translation 3–4 kb upstream, as it disrupts this essential long-distance interaction (Miller and White, 2006).

Readthrough of the CP stop codon of viruses from the *Luteoviridae* family appears to use a fourth class of *cis*-acting signals (Brown et al., 1996). The stop codon is usually UAG, but can be UGA or UAA. Instead, readthrough requires a tract of 8–16 repeats of CCXXXX beginning about 8 nt downstream of stop codon and requires additional sequence about 700–750 nt downstream in the coding region of the readthrough ORF, in the example of BYDV (Brown et al., 1996). Although the resulting CP-readthrough protein fusion is not essential for virus particle assembly or infectivity it is assembled into the virion and is required for persistent, circulative aphid transmission (Brault et al., 1995; Chay et al., 1996).

## Perspectives

This review provides an outlook of the vast diversity of non-canonical mechanisms that RNA viruses use to translate their RNAs. With some significant exceptions, knowledge is still superficial for a large number of cases. It would be highly desirable to obtain additional and deeper information on specific cases and mechanisms. For example, secondary structure data is available for only a few translation initiation elements (Wang et al., 2009a; Zuo et al., 2010; Nicholson and White, 2011; Kraft et al., 2013; Miras et al., 2014), and high-resolution three-dimensional structures are known only for the small H-type pseudoknot frameshift structures of the polero- and enamoviruses (Miller and Giedroc, 2010), and for plant translation factor eIF4E (Monzingo et al., 2007; Ashby et al., 2011). There is no structural data on bipartite or multipartite virus-host complexes. This represents a significant methodological challenge, but the current advancement of techniques like cryo-electron microscopy may significantly contribute to tackle it. Structural data would provide additional mechanistic insight and could contribute to uncover interacting regions with regulatory roles, providing molecular targets for intercepting productive host-virus interactions.

It is important to note that mutations in translation initiation factors that disrupt interactions with viral proteins or RNA might not only prevent infection in resistant varieties of susceptible host species but also contribute to non-host resistance. Mutations in viral factors conferring compatibility with translation initiation factors of otherwise non-host plants can contribute to broaden the host range of potyviruses (Calvo et al., 2014; Estevan et al., 2014; Svanella-Dumas et al., 2014) and a carmovirus (Nieto et al., 2011). Also, the development of techniques to monitor the translational dynamics based on fluorescent and optical methods could provide more complete pictures of how and where translation occurs.

The diversity of mechanisms is particularly striking when identified in a single viral RNA. BRV provides an example of this, with gRNAs carrying VPg, poly(A), IRES, and CITE (Karetnikov et al., 2006; Karetnikov and Lehto, 2008), and there are other viral RNAs for which multiplicity of *cis*-acting elements has been recognized, including MNSV (Miras et al., 2014) and PEMV2 (Gao et al., 2013, 2014). This multiplicity may exist for different reasons, including the use of different mechanisms during different steps of the infection cycle or to infect different hosts, or the overlapping of templates for transcription of mRNAs which, again, may be translated during different steps of the infection cycle and/or in different cellular environments. This brings us to various additional methodological aspects that may require attention for further development of this research field: On the one hand, the dissection of the infection cycle is still a difficult task for plant virologists, as there is a lack of experimental systems in which synchronous infections can be established. On the other hand, experimental systems appropriate for performing arrays of experiments covering biochemistry, genetics and cellular biology are also missing. For instance, wheat germ extract has been and still is very useful for biochemistry experiments, but genetics or cellular biology experiments are difficult using wheat as a host, because it is hexaploid and difficult to transform. In another example, *N. benthamiana* is an excellent host to perform cellular biology experiments, but its genetic tractability is rather poor, and *N. benthamiana* is not particularly advantageous for biochemistry experiments. In this regard, the preparation of translationally active extracts from evacuolated protoplasts (Murota et al., 2011) from different plant species may contribute to solve this problem, particularly if prepared from genetically tractable and microscopy amenable hosts such as Arabidopsis.

From the point of view of the cellular translational machinery and how viruses use it, the described diversity of translation mechanisms points toward the different ways that viruses use and control the basic translation machinery of the cell, but it also seems to point toward the existence of a diversity of associations of RNA and protein translation factors used for the uninfected cell to synthesize proteins from different mRNA populations under different micro-environmental conditions and/or subcellular locations. It is tempting to speculate that during evolution plant viruses may have adopted cellular preexisting mechanisms to translate their proteins; quite likely, there is a significant overlap between translation mechanisms of viral RNAs and translation of cellular mRNAs in uninfected cells under abiotic stress conditions (Spriggs et al., 2010), and viruses might be viewed as useful probes to uncover the cellular mechanisms of translation. In this regard, it has been shown that active plant virus replication associates with host gene shutoff (Wang and Maule, 1995; Aranda and Maule, 1998), but even during active replication there are host mRNAs which are over-expressed, suggesting common mechanisms for host and viral mRNA expression, including translation; structural data may provide important information on how these transcripts can recruit the host machinery efficiently during cellular shut-off. Interestingly, host mRNAs over-expressed during virus replication include stress response transcripts (Aranda et al., 1996) and, in fact, at least a maize HSP101 and ADH1 transcripts have been shown to contain IRES-like elements (Dinkova et al., 2005; Mardanova et al., 2008). Large screenings of the human genome revealed widespread identification of cap-independent translation elements located in the 5′-UTR and 3′-UTR of human transcripts, but their mode of regulation remains unknown (Weingarten-Gabbay et al., 2016). In plants, there are few reports on ribosome profiling under abiotic stresses such are drought, varying external light conditions or in response to reactive oxygen species (Liu et al., 2013; Benina et al., 2015; Lei et al., 2015), but not under viral infection conditions, limiting the identification of potential parallelisms.

Last but not least, the diversity of *cis*-acting translation elements identified in plant viruses may contribute to the design of tools for synthetic biology (Ogawa et al., 2017), and in vectors for the overexpression of proteins in biofactory cell-free systems, cell cultures, or whole plants (Fan et al., 2012), or, perhaps, in other organisms used for industrial overexpression of proteins, if mechanisms employed by plant viruses are universal or at least conserved in the species of interest.

## Author contributions

MM wrote Sections Intergenic Region Enhancers to Optimization of Viral mRNA Coding Capacity, WM, VT, and MA edited and added specific information to all sections, MA supervised MM writing and wrote Sections Introduction and Perspectives.

### Conflict of interest statement

The authors declare that the research was conducted in the absence of any commercial or financial relationships that could be construed as a potential conflict of interest.
